# Neuropsychiatric symptoms and specific cognitive domains in mild cognitive impairment

**DOI:** 10.1590/1980-5764-DN-2024-0187

**Published:** 2025-01-13

**Authors:** Lucas Luchesi Barnes, Lucas Francisco Botequio Mella, Francisca Daniele Ferreira Lopes, Amilton dos Santos, Paulo Dalgalarrondo

**Affiliations:** 1 Universidade Estadual de Campinas, Faculdade de Ciências Médicas, Departamento de Psiquiatria, Campinas SP, Brazil.

**Keywords:** Cognitive Dysfunction, Neurobehavioral Manifestations, Neuropsychological Tests, Disfunção Cognitiva, Manifestações Neurocomportamentais, Testes Neuropsicológicos

## Abstract

Neuropsychiatric symptoms (NPS) such as depression, anxiety, and apathy are present in mild cognitive impairment (MCI) and are risk factors for cognitive decline. However, the relationship between NPS and specific cognitive domains is less clear.

**Objective:**

This study aimed to assess whether there is a correlation between NPS and specific cognitive domains.

**Methods:**

A cross-sectional study which included 174 participants with MCI, aged 55 years or older. Differences in sociodemographic profile, neuroimaging, and neuropsychological tests between MCI participants with and without NPS were measured.

**Results:**

Participants with NPS had lower education and worse performance in attention tests and executive functions. Psychotic symptoms were correlated with deficits in visuospatial functions; irritability and agitation with inattention and deficit of inhibitory control; and depression with inattention.

**Conclusion:**

Correlations were found between some NPS with specific cognitive domains, especially psychotic symptoms, but also agitation, irritability, apathy, and depression.

## RESUMO.

Sintomas neuropsiquiátricos (SNP) como depressão, ansiedade e apatia estão presentes no comprometimento cognitivo leve (CCL) e são fatores de risco para o declínio cognitivo. No entanto, a relação entre SNP e domínios cognitivos específicos é menos clara.

**Objetivo:**

Este estudo teve como objetivo avaliar se há correlação entre SNP e domínios cognitivos específicos.

**Métodos:**

Estudo transversal que incluiu 174 participantes com CCL, com idade igual ou superior a 55 anos. Foram avaliadas diferenças quanto ao perfil sociodemográfico, neuroimagem e testes neuropsicológicos entre participantes com CCL com e sem SNP.

**Resultados:**

Os participantes com SNP apresentaram menor escolaridade e pior desempenho em testes de atenção e funções executivas. Sintomas psicóticos foram correlacionados com déficits nas funções visuoespaciais; irritabilidade e agitação com desatenção e déficit de controle inibitório; e depressão com desatenção.

**Conclusão:**

Foram encontradas correlações entre alguns SNP com domínios cognitivos específicos, especialmente sintomas psicóticos, mas também agitação, irritabilidade, apatia e depressão.

## INTRODUCTION

The diagnosis of dementia implies a threshold between normality and cognitive impairment, evident to the point of making it impossible for the individual to work, manage finances, shop, travel independently, prepare meals, and even take care of themselves^1^. However, the main diseases responsible for cognitive alterations, Alzheimer's disease (AD) and cerebrovascular disease (CVD), do not present as dementia from the beginning. They are established slowly and progressively, affecting the subject along a continuum, from when only molecular and cellular alterations of the nervous system are present, without the appearance of symptoms or cognitive decline (CD), to the frank deterioration of cognition, behavior, and functionality, with significant brain atrophy^2^.

The stage of CD before the onset of dementia is called mild cognitive impairment (MCI) and, according to the 5^th^ edition of the Diagnostic and Statistical Manual of Mental Disorders (DSM-V, 2013), is defined by the presence of cognitive alterations in one or more domains. These are measured by standardized neuropsychological tests and generally lead to interference in the individual's functionality in complex activities of daily living, but without causing disability or dependence. Projections of population aging and increased prevalence of dementia^3^ have made it necessary to find quicker and cheaper ways of identifying individuals at risk of CD and instruments that can serve as everyday clinical assessments^4^.

In addition to cognitive impairment, there are also behavioral changes associated with CD, also known as neuropsychiatric symptoms (NPS). These are behavioral, emotional, and psychopathological changes, which include various domains of symptoms, such as depression, anxiety, agitation, apathy, and others. They are present at all stages of CD and in practically all their etiologies.

Some studies indicate that NPS are present in 50 to 85% of individuals with MCI and that they may be predictors of conversion to dementia, especially depression, apathy, and sleep disorders^5–8^. Research on this topic raises questions about the relationship between NPS and the progression of CD^9^.

The relation between NPS and specific cognitive domains is unclear, and it could help map CD trajectories before the onset of dementia since it is easier to assess these symptoms in comparison with biomarkers in neuroimaging or cerebrospinal fluid. This study aims to assess whether there is a correlation between NPS and specific cognitive domains according to performance in neuropsychological tests to better differentiate groups of individuals with these characteristics in a Brazilian sample of MCI patients.

## METHODS

### Design

This is an observational study of patients who were being followed up in the cognitive health program called "Take Care of Your Memory" at the Community Health Center (CECOM) of the State University of Campinas (UNICAMP)^10^. The inclusion criteria for this program are:

- age 55 or older;
- complaining of memory decline or other cognitive domains, compared to their previous cognitive performance.

The exclusion criteria were:

- the diagnosis of dementia;
- the occurrence of other serious conditions that cause mental disorders, such as primary psychiatric diseases (severe depression, bipolar disorder, schizophrenia, drug addiction, autism); neurological conditions (uncontrolled epilepsy, brain lesions such as neoplasms, inflammatory or infectious diseases); and
- recent unstable systemic conditions including heart diseases or other disorders which may impact CNS function. Neurodegenerative disorders and chronic cerebrovascular conditions may be included.

The study participants were assessed for the presence of NPS, sociodemographic profile, cognitive screening tests, neuropsychological tests, and neuroimaging exams. Only patients diagnosed with MCI were selected for this study. They were then divided into two groups: with and without NPS, according to the Neuropsychiatric Inventory — Clinician Rating Scale (NPI-C). The absence of NPS was defined as a score of 0 on this scale, and the presence of NPS was defined as any score greater than 0.

### Mild cognitive impairment and dementia criteria

The criteria for dementia used were from the 5^th^ edition of the Diagnostic and Statistical Manual of Mental Disorders (DSM-5). On the other hand, the diagnosis of MCI is based on the Revised Mayo Clinic Criteria for MCI (2003). Patients with a performance below 1.5 standard deviations on one or more of the tests in our battery were found to have cognitive impairment and therefore MCI.

### Cognitive assessments

The cognitive screening instruments used were the Mini-Mental State Examination (MMSE)^11^ and the Montreal Cognitive Assessment (MoCA)^12–14^. The neuropsychological tests consisted of a predefined battery of cognitive tests. The tests carried out were: Rey's Auditory-Verbal Learning Test (RAVLT), which assesses recent memory and learning; the Digit Span tests, related to attention and working memory; the Stroop Color Tests, which measures executive functions and inhibitory control; phonemic verbal fluency tests (words beginning with "F", "A" and "S") and semantic verbal fluency tests (animal names), which evaluates executive functions and language; Luria's Visuospatial Perception test, related to visuospatial functions; Rey-Osterrieth Complex Figure (ROCF), which looks at executive functions, visuospatial functions, and visual memory; Trail Making Test A (TMT-A) and B (TMT-B), which refer to executive functions and cognitive processing speed; and the Boston Naming Test (BNT), which investigates language^15–25^.

### Neuropsychiatric symptoms assessment

The questionnaire used in this study to measure neuropsychiatric symptoms was the Neuropsychiatric Inventory - Clinician Rating Scale (NPI-C). The original version of this instrument, the Neuropsychiatric Inventory (NPI), is most used in the assessment of neuropsychiatric symptoms both in research and in clinical practice, and its completion is based on the responses of the caregiver or family member who lives with the patient^26^. However, the NPI-C considers the impression of the patient and the clinician as well. It was therefore more interesting for this research, where the patient was often unaccompanied. In addition, the new version has revised the neuropsychiatric domains, improved the reliability of the domains separately, and deepened the evaluation of each domain. The neuropsychiatric domains established in the NPI-C are delusions, hallucinations, agitation, aggressiveness, depression/dysphoria, anxiety, elation/euphoria, apathy/indifference, disinhibition, irritability/lability, aberrant motor disorder, sleep disorders, appetite and eating disorders, and aberrant vocalizations^27^.

### Neuroimaging

Neuroimaging scans (nuclear magnetic resonance or computed tomography of the brain, without contrast) assessed gross global alterations (such as neoplasms, infections, and macrovascular alterations) and general cortical atrophy. More attention was paid to assessing the atrophy of the hippocampus and entorhinal cortex due to the relationship between atrophy of this region and Alzheimer's disease^28^. Duara scale or medial temporal lobe atrophy (MTA)^29^ was used for this purpose. The degree of cerebral microangiopathy was measured using the Fazekas visual scale. Its score is from 0 to 3, with 0 (zero) being the absence of microangiopathy, 1 (one) mild microangiopathy (non-confluent foci), 2 (two) moderate microangiopathy (local confluences), and 3 (three) severe microangiopathy (confluences between different regions)^30^.

### Ethics considerations and consent statement

This study only used data from the medical records of patients followed up by the "Take Care of Your Memory" Program at CECOM/UNICAMP^10^, whose care and assessment protocols are primarily for care purposes. The patients were not re-submitted to interviews, assessments, tests, or laboratory and neuroimaging scans, therefore new consent was not needed. This study was submitted to the Research Ethics Committee of the Faculty of Medical Sciences of UNICAMP, under Certificate of Presentation for Ethical Consideration — CAAE 45739515.7.0000.5404, and was approved.

### Analyses of results

The Mann-Whitney U-test was used to compare groups in terms of continuous numerical variables and Pearson's chi-square (χ^2^) test for categorical variables. The socio-demographic data analyzed were age, education level, gender, personal history of smoking, hypertension, diabetes mellitus, and dyslipidemia. Median scores for MTA and Fazekas scales were also compared. Cognitive tests were compared individually with the groups with or without NPS. Subsequently, the correlation between neuropsychiatric symptoms and cognitive domains was assessed using Spearman's correlation test. The data was analyzed using Statistical Package for the Social Sciences — SPSS (Chicago, IL, USA) version 25. A p<0.05 was considered statistically significant.

## RESULTS

During the collection period, 269 patients entered the program, according to the inclusion criteria. Throughout the initial assessment protocol, 23 dropped out and did not complete the evaluation. Twenty-six met the exclusion criteria, were excluded from the analyses, and were referred to appropriate services (14 with dementia, seven with severe depression, two with schizophrenia, two with attention deficit hyperactivity disorder, and one with severe bipolar disorder). The sample analyzed consisted of 220 individuals with cognitive complaints and no dementia. MCI was diagnosed in 174 participants (79.1%) and, of these, 81 (46.5%) did not have NPS compared to 93 (53.5%).

Among the patients without NPS, there were 65 females (80.2%) and 16 males (19.8%), while in the group with NPS 74 were female (79.6%) and 19 were male (20.4%), so there was no difference between the two groups in this aspect (p=0.912). The average age and education level in years were 62.3 and 11.8 in the group without NPS and 63.2 and 10.3 in the group with NPS, respectively. There was a significant difference between the groups only with regard to education level, which was lower in the NPS group (Table 1).

**Table 1:** Sociodemographic Data, Screening Tests and Neuroimaging Scales.

	Neuropsychiatric symptoms		Test value	p-value
	Absent	Present		
Age (mean, SD)	62.3 (7.5)	63.2 (5.8)	4371.5*	0.067
Education (years, SD)	11.8 (4.2)	10.3 (4.4)	2917.5*	**0.010**
Female (%)	65 (80.2)	74 (79.6)	0.012 (1)†	0.912
Tobacco use (%)	12 (14.8)	24 (25.8)	3,188 (1)†	0.074
Hypertension (%)	42 (51.9)	49 (52.7)	0.12 (1)†	0.912
DM (%)	30 (37)	32 (34.4)	0.13 (1)†	0.718
Dyslipidemia (%)	34 (42)	43 (46.2)	0.319 (1)†	0.572
Fazekas (mean, SD)	1,0 (0.6)	1,0 (0.7)	2862*	0.853
MTA (mean, SD)	0,3 (0.5)	0,4 (0.6)	2559*	0.171
MMSE (mean, SD)	26.8 (2.2)	26.9 (2.0)	3783.5*	0.932
MoCA (mean, SD)	23.0 (3.5)	22,4. (4.1)	3539*	0.491

Abbreviations: SD, standard deviation; DM, diabetes mellitus; MTA, medial temporal lobe atrophy; MMSE, Mini-Mental State Examination; MoCA, Montreal Cognitive Assessment.

Notes:

* Mann-Whitney U Test;

† Pearson's Chi-squared test (degree of freedom).

Bold indicates p-values<0.05.

Regarding personal history, in the group without NPS, 14.8% were smokers, 51.9% hypertensive, 37% diabetic and 42% dyslipidemic; in the group with NPS, 25.8% were smokers, 52.7% hypertensive, 34.4% diabetic, and 46.2% dyslipidemic. There was no difference between the two groups in terms of the presence of any of these antecedents (Table 1).

The Fazekas and MTA visual scales also showed no differences between the groups. On the former, 71 (87.7%) of the individuals without NPS and 77 (82.8%) of the individuals with NPS scored above 0, with a Fazekas scale median score of 1.0 (standard deviation — SD=0.6) and 1.0 (SD=0.7), respectively (p=0.853). In the latter, 32 (39.5%) of the patients in the group without NPS and 45 (48.4%) of the patients in the group with NPS scored more than 0, with an MTA median score of 0,3 (SD=0.5) and 0,4 (SD=0.6), respectively (p=0.171).

Concerning the cognitive screening tests, MMSE and MoCA, the groups with and without NPS also had no statistically significant difference (p=0.932; p=0.941, respectively), despite a slightly lower total score on the MoCA for the group with NPS, 22.4 for the group with NPS and 23.0 for the group without NPS (Table 1).

The frequency of NPS and their means and SD are shown in Table 2. The NPS domains with the highest frequencies were, in descending order: depression/dysphoria (67%), sleep disorders (59%), anxiety (41%), irritability (30%), apathy (26%), agitation (10%), appetite and eating disorders (9%), hallucinations (3%) and delusions (2%). The NPS domains with the highest mean scores were, in descending order: depression/dysphoria (4.0), anxiety (3.1), apathy/indifference (2.8), irritability/lability (1.9), sleep disorders (1.8), delusions (0.3), agitation (0.2), appetite and eating disorders (0.2) and hallucinations (0.05). The average total score on the NPI-C scale was 14.2. The domains aggression, euphoria/elation, disinhibition, aberrant motor disturbance, and aberrant vocalizations scored 0.

**Table 2:** Total score and score measures per neuropsychiatric domain measured by the Neuropsychiatric Inventory – Clinician Rating Scale.

DOMAIN	n (%)	Mean (SD)
Delusion	2 (2)	0.3 (0.2)
Hallucinations	3 (3)	0.05 (0.3)
Agitation	9 (10)	0.21 (0.7)
Aggressiveness	0	0
Depression/Dysphoria	62 (67)	4.0 (4.3)
Anxiety	38 (41)	3.1 (4.6)
Euphoria/Elation	0	0
Apathy/Indifference	24 (26)	2.8 (5.3)
Disinhibition	0	0
Irritability/Lability	28 (30)	1.9 (3.6)
Aberrant Motor Disorder	0	0
Sleep Disorders	54 (59)	1.8 (2.2)
Appetite and Eating Disorders	8 (9)	0.2 (0.6)
Aberrant Vocalizations	0	0
NPI-C total	93	14.2 (10.7)

The comparison of performance in neuropsychological tests between the two groups showed that the presence of NPS was positively associated with worse performance in the time measure of the Stroop Color Test Congruent, TMT A, and B (Table 3). No correlation was found between age and education level with NPS as a whole or with specific neuropsychiatric domains (Table 4).

**Table 3:** Comparison between groups with and without neuropsychiatric symptoms on specific neuropsychological tests.

	Neuropsychiatric symptoms		Mann-Whitney U Test	p-value
	Absent	Present		
RAVLT- LOT	15.6 (6.5)	15.5 (7.1)	3,628	0.676
RAVLT- Delayed Recall	8.7 (3.1)	8.0 (3.0)	3,506.5	0.431
RAVLT- Recognition	9.9 (5.2)	10.1 (4.1)	3,788.5	0.947
Forward Digit Span	4.6 (1.0)	4.4 (0.8)	3,321.5	0.195
Reverse Digit Span	3.7 (1.0)	3.4 (1.1)	3,234	0.124
Stroop Color Test Congruent (time)	43.0 (11.0)	45.9 (11.4)	4,350.5	0.055
Stroop Color Test Congruent (errors)	0.1 (0.4)	0.04 (0.2)	3,679.5	0.806
Stroop Color Test Incongruent (time)	113.0 (45.8)	141.3 (56.5)	4,576	**0.002**
Stroop Color Test Incongruent (errors)	2.3 (3.2)	2.8 (4.1)	3,869.5	0.361
ROCF – Copy	26.6 (9.5)	25.2 (8.7)	3,180.5	0.077
ROCF - Memory	9.0 (5.5)	9.7 (6.1)	3,598	0.892
Semantic Verbal Fluency	15.0 (3.7)	14.2 (4.2)	3,277	0.138
Phonemic Verbal Fluency	30.1 (10.8)	27.4 (9.2)	3,302.5	0.161
Luria's Figures	13.1 (1.6)	12.8 (1.9)	3,429	0.300
TMT A	74.2 (31.0)	89 (45)	4,532	**0.014**
TMT B	144.2 (74.5)	210 (167.5)	3,913	**0.012**
TNB	54 (5.3)	53 (5.7)	3,383.5	0.247

Abbreviations: BNT, Boston Naming Test; LOT, learning over trials; ROCF, Rey-Osterrieth Complex Figure; RAVLT, Rey Auditory Verbal Learning Test; TMT, Trail Making Test. Bold indicates Notes: Bold indicates p-values<0.05.

**Table 4:** Spearman's correlation between neuropsychiatric symptoms, age, education.

	1	2	3	4	5	6	7	8	9	10
1. Age										
2. Education	**-0.349** *									
3. Delusions	0.015	-0.069								
4. Hallucinations	-0,060	-0.090	**0.817** *							
5. Agitation	-0.021	-0.141	-0.049	-0.060						
6. Depression/Dysphoria	-0.136	0.068	0.147	0.050	-0.182					
7. Anxiety	-0.186	0.044	0.034	-0.021	**0.365** *	-0.011				
8. Apathy/Indifference	-0.040	-0.029	**0.235** †	0.158	-0.082	**0.367** *	-0.089			
9. Irritability/Lability	0.113	-0.118	-0.096	0.004	**0.269** *	-0.105	**0.297** *	-0.135		
10. Sleep disorders	-0.046	0.012	-0.016	0.019	-0.051	0.119	0.033	0.133	0.045	
11. Appetite and eating disorders	-0.143	0.046	-0.046	-0.57	-0.101	**0.215** †	0.104	**0.284** *	0.008	0.146

Abbreviation: NPI-C: Neuropsychiatric Inventory – Clinician.

Notes:

* p<0.01;

† p<0.0. Spearman's correlation coefficient classification: 0.6<p<1.0=strong; 0.4<p<0.6=moderate; 0.2<p<0.4=weak.

Bold indicates p-values<0.05.

However, there was a negative correlation between age and education level, indicating that the higher the age, the lower the education level (p=-0.349; p<0.01). Education level was negatively correlated with the number of cerebrovascular risk factors (smoking, hypertension, dyslipidemia, and diabetes mellitus), indicating that the higher the education level, the lower the number of cerebrovascular risk factors (p=2.81; p<0.01). That said, the number of cerebrovascular risk factors was positively correlated with apathy/indifference (p=0.232; p<0.05). Some NPS domains showed a correlation with each other: delusions correlated positively with hallucinations (p=0.817; p<0.01) and apathy (p=0.235; p<0.05); agitation correlated positively with anxiety (p=0.365; p<0.01) and irritability (p=0.269; p<0.01); depression correlated positively with apathy (p=0.367; p<0.01) and eating and appetite disorders (p=0.215; p<0.05); anxiety was positively correlated with irritability (p=0.297; p<0.01); and apathy was positively correlated with eating and appetite disorders (p=0.284; p<0.01).

Among the tests that assess attention and executive functions, a negative correlation was found between irritability and Forward Digit Span Test (p=-0.263; p<0.05); between delusions and RALVT-Recognition (p=-0.255; p<0.05), Reverse Digit Span (p=-0.377; p<0.01), Luria's Figures (p=-0.268; p<0.05) and BNT (p=-0.345; p<0.01); and between hallucinations with Reverse Digit Span (p=-0.386; p<0.01), ROCF-Copy (p=0.232; p<0.05), Luria's Figures (p=-0.263; p<0.05) and BNT (p=-0.282; p<0.05). In tests related to attention and inhibitory control, a positive correlation was found between agitation and the number of errors in the Stroop Color Test Congruent (p=0.285; p<0.01). Depression had a positive correlation with time measure in the Stroop Color Test Incongruent (p=0.283; p<0.01) Table 5.

**Table 5:** Spearman's correlation between neuropsychiatric symptoms and neuropsychological tests.

	MMSE	MoCA	RAVLT-LOT	RAVLT- Delayed Recall	RAVLT-Recognition	Forward Digit Span	Reverse Digit Span	TMT A	TMT B	Stroop Color Test Congruent (time)	Stroop Color Test Congruent (errors)	Stroop Color Test Incongruent (time)	Stroop Color Test Incongruent (errors)	ROCF - Copy	ROCF - Memory	Luria's Figures	Semantic Verbal Fluency	Phonemic Verbal Fluency	BNT
NPI-C total	-0.017	-0.106	0.072	-0.034	0.142	-0.112	-0.039	-0.018	-0.069	-0.066	-0.066	0.029	0.029	-0.046	-0.131	0.117	0.135	0.012	-0.052
Delusions	-0.202	-0.136	0.063	-0.215	**-0.225** *	0.025	**-0.377** †	-0.017	-0.105	-0.067	-0.017	0.062	0.081	-0.196	-0.089	**-0.268** *	-0.099	0.021	**-0.345** †
Hallucinations	-0.201	-0.152	0.143	-0.158	-0.209	-0.010	**-0.386** †	-0. 019	-0.131	0.005	-0.038	0.010	0.048	**-0.232** *	-0.076	**-0.263** *	-0.125	0.068	**-0.282** *
Agitation	-0.039	-0.073	-0.031	-0.031	-0.119	0.143	-0.069	-0.011	-0.074	-0.025	**0.285** †	-0.043	-0.015	-0.069	0.043	0.115	0.034	-0.020	0.026
Depression	-0,151	-0,104	0,153	-0,002	0,043	-0,162	-0,029	0,093	0,043	0,065	-0,133	**0,283** †	0,034	-0,025	-0,110	0,066	0,093	-0,001	-0,112
Anxiety	0.004	0.021	0.075	0.064	0.083	0.066	0.186	-0.026	0.007	-0.081	0.135	-0.101	0.060	0.027	0.811	-0.073	0.861	0.073	-0.006
Apathy	-0.010	-0.117	-0.139	0.141	0.019	-0.031	-0.133	-0.090	-0.118	0.038	-0.108	0.019	-0.127	-0.001	-0.096	0.135	0.145	0.003	-0.057
Irritability	0.059	-0.078	-0.043	0.009	0.142	**-0.263** *	-0.092	0.048	-0.011	-0.129	-0.065	-0.100	0.210	-0.059	-0.039	0.103	0.009	-0.066	0.079
Sleep disorders	0.150	0.122	0.091	-0.031	0.173	0.054	0.075	-0.002	0.012	0.013	-0.027	-0.026	-0.051	-0.017	-0.198	0.088	-0.093	-0.028	0.057
Appetite disorders	0.081	-0.072	0.049	-0.059	0.188	0.114	0.047	-0.211	-0.119	-0.110	-0.57	-0.040	-0.130	-0.059	-0.090	0.013	-0.068	0.069	-0.048

Abbreviations: BNT, Boston Naming Test; LOT, learning over trials; MMSE, Mini-Mental State Examination; MoCA, Montreal Cognitive Assessment; NPI-C, Neuropsychiatric Inventory – Clinician; RAVLT, Rey Auditory Verbal Learning Test; ROCF, Rey-Osterrieth Complex Figure; TMT, Trail Making Test.

Notes:

* p<0.05;

† p<0.01.

Classification of the Spearman correlation coefficient, 0.6<p<1.0=strong; 0.4<p<0.6=moderate; 0.2<p<0.4=weak. Bold indicates p-values<0.05.

## DISCUSSION

The prevalence of NPS found in patients with MCI was 53.5%, which is compatible with the average found in the literature, which varies between 35-85%^7^. Patients with NPS had fewer years of educational level than patients without NPS. Educational level is a known protective factor for CD^3,31^. However, there may be a relationship between educational level and NPS in patients with CD, as demonstrated in a meta-analysis that assessed the prevalence of NPS in patients with Alzheimer's disease. In this study, apathy was the most common NPS and was associated with greater severity of CD and was influenced by low educational level^25,32^.

The presence of white matter lesions (WML) in the brain can be related to cognitive and neuropsychiatric alterations^33^. However, there was no statistical difference between the groups with and without NPS concerning changes in the presence of microangiopathy (p=0.853). Most patients with MCI had some degree of microangiopathy alteration on neuroimaging (more than 80% in both groups), consistent with a systematic review of the literature which found that between 75 and 100% of patients with MCI had some degree of microangiopathy^34^. Nonetheless, it was higher than another study with a similar patient profile^35^. One possible explanation for this is the higher prevalence of DM, hypertension, dyslipidemia, and smoking in this sample than in the general Brazilian population^36–38^. Thus, WML were not able to differentiate patients with MCI about the presence or absence of NPS and explain the difference between these two groups in some tests that assess executive functions and attention, such as the Stroop Color Test, TMT A, and B.

According to the correlation data in Table 4, age and educational level did not correlate with NPS, but they did correlate negatively with each other. This reflects the lower educational level of the elderly in Brazil while demonstrating that the population has become more educationalized in recent decades. This is a risk factor for dementia, which is responsible for up to 7%^3^ of modifiable risk factors and there is still room in Brazil for change^39^.

Furthermore, according to the data in Table 4, NPS can be divided into 3 groups: delusions and hallucinations; anxiety, agitation, and irritability; depression, apathy and eating and appetite disorders. This grouping is similar to a survey on the profile of NPS in MCI patients from a larger sample (n=187) in Brazil^40^ and has little difference from other possible clusters. One of these other models would be with a cluster of hyperactivity symptoms (agitation, irritability, and disinhibition), a cluster of affective symptoms (depression, apathy, sleep, anxiety, and eating and appetite disorders), and a cluster of psychotic symptoms (delusions and hallucinations)^41^.

In comparison to dementia stages, another study identified four neuropsychiatric subsyndromes in a large sample of outpatients with Alzheimer's Dementia: hyperactivity, psychosis, affective symptoms, and apathy. Apathy subsyndrome was the most common (65%) while the psychosis subsyndrome was less prevalent (38%), but was associated with the highest level of total neuropsychiatric problems^42^. This similarity of clusters shows that, probably, there is a continuum of clusters of NPS that initiates before dementia and continues throughout the disease course and may be used as a prognostic tool^43^. It may also reflect specific neurocircuits that are compromised and may be correlated with specific cognitive symptoms.

In our study, analyzing the first cluster, delusions, and hallucinations correlated negatively with attention and working memory (Reverse Digits Span Test), visuospatial functions (Luria's Figures Test), and language (BNT); delusions also correlated negatively with memory (RAVLT-Delayed Recall). Psychotic symptoms, especially hallucinations, may be present as a prodrome of some neurodegenerative diseases such as Lewy Body Dementia and are more associated with inattention and visuospatial deficit^44^.

The second cluster had a negative correlation between irritability and attention (Forward Digits Span Test) and agitation and inhibitory control (Stroop Color Test Incongruent). These symptoms may be more related to dysfunction in the orbitofrontal-subcortical circuits, anterior cingulate gyrus, amygdala, and hippocampus, which are mainly associated with attention, executive functions, and memory^45^.

Within this third cluster, only depressive symptoms were positively correlated with attention and inhibitory control (Stroop Incongruent), while apathy and eating and appetite disorders did not correlate with any cognitive domain in this sample. Depressive symptoms are the most prevalent SNP in samples of patients with MCI and are associated with reduced hippocampal volume, especially in Alzheimer's disease, but may also be related to WML^45^.

The findings of this study point to the existence of a relationship between some NPS domains and between these and some cognitive domains in a more specific way. In clinical practice, this could lead to more targeted rehabilitation and treatment. From a research point of view, it could also lead to a better selection of patients for studies into the pathophysiology and treatment of MCI.

The limitations of this study include the small number of individuals in the groups with and without MCI, the impossibility of separating patients with biomarkers for Alzheimer's disease, and the use of the NPI scale for patients with MCI since this scale has been validated for dementia. In addition, the analysis of the impact of NPS on neuropsychological tests through continuous measurement may have been compromised, since the use of measures such as Z-score may be more interesting for this purpose.

We aimed to investigate potential associations between neuropsychiatric symptoms (NPS) and specific cognitive domains, focusing on discovering patterns that warrant further investigation. Based on the analysis of the data presented, there are differences between patients with and without NPS in terms of cognitive profile, but there are also differences between the relationships of NPS with each other and with specific cognitive domains. This distinction is important in trying to clarify the relationships between NPS and CD in the attempt to identify and select individuals for analysis and treatment. It may also help to identify brain circuits that could contribute to a better understanding of the pathophysiology of NPS and CD and potential treatment targets.
